# Numerical Optimization and Experimental Validation of Finite Perforated Cellular Panels for Vibration Reduction

**DOI:** 10.3390/ma18245620

**Published:** 2025-12-15

**Authors:** Bastián Sáez, Viviana Meruane, Rubén Fernández, Erick I. Saavedra Flores

**Affiliations:** 1 Department of Mechanical Engineering, University of Chile, Santiago 8370456, Chile; bastian.saez.a@ug.uchile.cl (B.S.); rufernan@uchile.cl (R.F.); 2 Department of Civil Engineering, University of Santiago of Chile, Av. Víctor Jara 3659, Santiago 9170124, Chile; erick.saavedra@usach.cl

**Keywords:** finite perforated panels, vibration mitigation, cellular structures, Particle Swarm Optimization (PSO), frequency response function (FRF)

## Abstract

Mechanical vibrations in lightweight structures remain a persistent challenge, often leading to noise, fatigue, and performance degradation in aerospace, automotive, and industrial applications. Recent advances in phononic crystals and perforated metaplates have shown that periodic cavities or uniformly distributed perforations can generate bandgaps and reduce vibration transmission. However, most existing designs rely on identical and regularly spaced holes, which limits the ability to precisely tune the attenuation response. This work introduces a novel design and optimization framework for finite perforated cellular panels, in which each perforation diameter is individually optimized to achieve targeted vibration suppression within specific frequency ranges. Finite element models were coupled with a Particle Swarm Optimization (PSO) algorithm to minimize the frequency response function (FRF) amplitude. Aluminum panels with 16 and 25 perforations were optimized, fabricated via CNC machining, and experimentally validated using impact hammer tests. The optimized designs achieved up to 90% reduction in vibrational amplitude within the target frequency bands, demonstrating strong agreement between numerical predictions and experimental results. These results highlight the potential of non-periodic, locally optimized perforation patterns as a practical and scalable approach for vibration control in finite structural components.

## 1. Introduction

Lightweight engineering structures are widely used in aerospace, automotive, and industrial applications, where undesired vibrations can lead to noise, fatigue, and performance loss. Traditional approaches for vibration control commonly rely on additional damping layers or isolation systems. While effective, these solutions often increase weight and cost, which is undesirable in weight-sensitive sectors such as transportation and aeronautics.

An alternative strategy is to modify the structural geometry by introducing perforations or periodic features that alter the dynamic response of the system. Perforated plates, in particular, have been extensively studied in acoustics and vibration research as a practical means to attenuate structural vibrations and reduce sound radiation. The introduction of holes can redistribute stiffness and mass, creating local changes in modal behavior and, in some cases, effective attenuation of vibrational energy [1]. Early works demonstrated that perforations influence both stiffness and mobility of thin plates, highlighting the need for accurate modeling of their dynamic properties [2]. More recent studies extended this analysis to partially perforated geometries, such as circular plates, confirming that perforations significantly affect natural frequencies and modal distributions [3]. Within this framework, perforated and phononic plates have emerged as promising candidates for lightweight and cost-efficient vibration suppression solutions, since they exploit geometric discontinuities to induce scattering and interference effects that reduce vibration transmission.

Most of the literature on phononic and metamaterial plates assumes idealized infinite periodicity, where the structure is represented by a repeating unit cell subjected to Bloch–Floquet boundary conditions. These models provide valuable insights into wave dispersion and bandgap mechanisms but neglect the finite-size effects, boundary conditions, and modal coupling that dominate in practical engineering components. In real applications, structures such as panels, casings, or enclosures are inherently finite, and their vibrational response cannot be captured solely through periodic analyses.

Recent works have demonstrated that even in finite perforated plates, geometric discontinuities can lead to localized resonance and partial attenuation effects similar to those observed in infinite periodic media. However, these effects are highly dependent on the actual plate dimensions, boundary conditions, and hole distribution, motivating the need for explicit modeling and optimization of finite systems.

Periodic perforated plates and phononic crystals have been widely investigated for their ability to inhibit wave propagation in certain frequency ranges, known as bandgaps. These structures achieve vibration suppression by exploiting periodic cavities or inclusions that alter dispersion properties and induce destructive interference effects. For instance, Andreassen et al. demonstrated directional bending wave propagation in periodically perforated plates, showing how periodic voids can create both partial and full bandgaps with anisotropic wave behavior [4]. Similarly, Tomita et al. analyzed elastic wave attenuation in metaplates with periodic hollow shapes, demonstrating experimentally that such geometries generate effective bandgaps for vibration suppression [5]. Wang et al. investigated phononic crystals with cross-like holes, reporting that non-convex geometries enable the formation of large and tunable bandgaps at relatively low frequencies [6].

Advanced geometric configurations have also been proposed to broaden attenuation zones. Huang et al. studied slabs with cross-like holes on oblique lattices and showed that multiple flexural-wave attenuation zones can be achieved, with good agreement between numerical and experimental results [7]. Das et al. extended this approach to plates with periodic cavities of varying shapes (square, circular, and rectangular), confirming the sensitivity of bandgap formation to cavity geometry and validating their predictions through experimental testing [8].

Other investigations have examined acoustic scattering and dynamic effects in perforated plates. Cavalieri et al. developed a numerical framework for acoustic scattering by finite perforated elastic plates, highlighting how elasticity and porosity jointly influence the scattered field and vibration–acoustic coupling [9]. Carbajo et al. introduced a finite element model for perforated panel absorbers that accounts for viscothermal effects, enabling more accurate representation of acoustic losses in complex perforated configurations [10]. Similarly, Smirnov and Lebedev analyzed free vibrations of perforated thin plates with multiple cut-outs, emphasizing how hole size, shape, and position affect modal properties and natural frequencies [11].

Together, these studies establish that periodicity and geometric complexity play a central role in the attenuation of structural vibrations and acoustic scattering. However, most works focus on uniform or repeated perforation patterns in infinite or quasi-infinite domains, leaving limited flexibility to fine-tune the response in finite, bounded structures.

Recent research has therefore shifted toward expanding the attenuation capabilities of perforated plates by moving beyond uniform hole configurations. One promising approach is the use of multiple hole diameters within the same structure. Kim and Yoon proposed a perforated plate with multiple-sized holes separated by porous partitions, showing that such configurations can substantially extend the absorption bandwidth compared to conventional single-sized designs [12]. Similarly, Gallerand et al. demonstrated that microperforated plates with multi-size perforations and optimized spatial distributions can provide enhanced damping over a broader frequency range, especially when perforations are located in regions of maximum modal displacement [13]. These works confirm that combining perforations of different sizes allows multiple resonance mechanisms to coexist, thereby broadening the effective attenuation band.

Parallel to these advances, optimization techniques have been applied to perforated and composite plates to enhance vibration performance. Duan introduced a two-dimensional sampling optimization method for composite plates with multiple circular holes, maximizing their fundamental frequency under various boundary conditions [14]. Complementing this, Mohammadi et al. reviewed micro-perforated panel structures, identifying perforation diameter, perforation ratio, panel thickness, and spatial distribution as the most influential design parameters [15]. These studies highlight that precise control and optimization of geometric features are crucial for achieving wideband and efficient vibration attenuation.

Despite the significant progress made in understanding the dynamic behavior of perforated and phononic plates, most existing studies have focused on identical perforations or grouped subsets with uniform sizes. While effective in generating bandgaps, these designs limit flexibility and the ability to finely tune attenuation within specific frequency ranges. Although advanced optimization methods, such as topology optimization and sampling-based algorithms, have been applied, they typically target global parameters or hole arrangements without addressing the possibility of treating each perforation as an independent variable. To date, the optimization of each perforation diameter individually has not been systematically explored. This gap highlights the opportunity to achieve more precise tailoring of the vibrational response by leveraging hole-to-hole variability within a single, finite plate.

Optimizing perforated panels is inherently a nonlinear and multimodal problem, as changes in individual hole diameters simultaneously affect local stiffness, mass distribution, and modal coupling. Heuristic and population-based algorithms, such as Particle Swarm Optimization (PSO), have emerged as powerful tools to address this class of problems due to their robustness, simplicity, and ability to escape local minima. Since its introduction by Kennedy and Eberhart [16], PSO has gained broad adoption because of its conceptual simplicity, small number of control parameters, and suitability for parallel computation. The method balances exploration and exploitation through stochastic updates of particle velocities and positions, guided by both personal and global best solutions [17,18]. Over the years, several variants, such as inertia-weighted and constriction-factor PSO, have improved convergence stability and search efficiency [19,20]. Comparative studies have shown that PSO performs particularly well in continuous optimization problems with bounded design variables, including geometric parameter tuning in structural and acoustic applications [21]. In the present work, PSO is employed to minimize the root-mean-square magnitude of the frequency response function (FRF) within prescribed frequency bands, enabling vibration suppression through direct geometric optimization without requiring gradient information.

The present study therefore introduces a novel methodology that combines finite element modeling (FEM) with Particle Swarm Optimization (PSO) to optimize the diameter of each perforation individually, targeting vibration suppression within predefined frequency ranges. This work focuses on the case of a finite perforated panels where modal behavior, boundary effects, and hole-to-hole interactions play a dominant role. The proposed numerical framework is validated experimentally through impact-hammer testing of aluminum panels fabricated via CNC machining. The results demonstrate that individualized perforation optimization can achieve vibration reductions of up to 90% in selected bands, clearly outperforming traditional uniform or periodic perforation designs. Beyond its scientific novelty, the proposed approach offers a lightweight, scalable, and cost-effective solution for vibration control, with promising applications in aerospace, automotive, and industrial systems where structural vibration mitigation is critical.

## 2. Background

### 2.1. Frequency Response Functions (FRF)

The frequency response function (FRF) links dynamic excitation and structural response in linear vibroacoustics. For a multi-degree-of-freedom structure, the equations of motion read

(1) $$M\;\ddot{u}\left(t\right)+C\;\dot{u}\left(t\right)+K\;u\left(t\right)=F\left(t\right),$$

where M, C and K are the mass, damping and stiffness matrices, respectively, and F(t) is the external force vector as a function of time *t*. u(t) denotes the displacement vector, $\dot{u}\left(t\right)$ and $\ddot{u}\left(t\right)$ its velocity and acceleration vectors, respectively.

Passing to the frequency domain gives the dynamic stiffness matrix

(2) $$Z\left(\omega \right)=-\omega^{2}M+j\;\omega \;C+K,$$

with $j=\sqrt{-1}$ the imaginary unit and ω=2πf the angular frequency associated with the frequency *f* (in Hz).

The Fourier transform of the time-domain quantities is denoted by a hat, so that $\hat{u}\left(\omega \right)=F\left\{u\left(t\right)\right\}$ and $\hat{F}\left(\omega \right)=F\left\{F\left(t\right)\right\}$ represent the displacement and force vectors in the frequency domain. Similarly, the acceleration in the frequency domain is given by $\hat{a}\left(\omega \right)=-\omega^{2}\hat{u}\left(\omega \right)$. The relationship between excitation and response can therefore be written as $\hat{a}\left(\omega \right)=Z^{-1}\left(\omega \right)\hat{F}\left(\omega \right)$, where Z(ω) is the dynamic stiffness matrix defined above.

Let $H_{ik}\left(\omega \right)$ denote the FRF between excitation degree of freedom (DOF) *k* and response DOF *i*. In the experiments and figures we work with accelerance, i.e., the magnitude of acceleration per unit force,

(3) $$H_{ik}\left(\omega \right)\equiv \left|\frac{{\hat{a}}_{i}\left(\omega \right)}{{\hat{F}}_{k}\left(\omega \right)}\right|=\omega^{2}\left|\frac{{\hat{u}}_{i}\left(\omega \right)}{{\hat{F}}_{k}\left(\omega \right)}\right|.$$

Here, ${\hat{a}}_{i}\left(\omega \right)$ and ${\hat{F}}_{k}\left(\omega \right)$ are the Fourier transforms of the acceleration $a_{i}\left(t\right)={\ddot{u}}_{i}\left(t\right)$ and excitation force $F_{k}\left(t\right)$ applied at DOFs *i* and *k*, respectively. Accordingly, $H_{ik}\left(\omega \right)$ represents the frequency-dependent transfer function between the input force and the acceleration response at the measured locations, expressed as magnitude of acceleration per unit excitation force.

For notational simplicity we refer to H(·) as the FRF throughout. Resonances appear as local maxima of *H*, whereas antiresonances and attenuation zones correspond to minima. When periodic microstructures are present, multiple scattering and local resonances can yield frequency intervals of reduced bending-wave transmission (band gaps), which manifest as sustained depressions in *H* over a band [22,23].

Because several response locations are measured numerically and experimentally, we form a location-averaged FRF magnitude

(4) $$\bar{H}\left(f\right)\;=\;\frac{1}{N_{r}}\sum_{i=1}^{N_{r}}H_{ik^{*}}\left(2\pi f\right),$$

where $N_{r}$ is the number of response DOFs (or sensor locations) and $k^{*}$ is the selected excitation DOF (hammer impact point) in a single-input/single-output setting. The overbar indicates averaging across response positions, providing a global measure of the panel’s dynamic response to a single excitation point.

The optimization is posed over prescribed frequency bands $B_{f}=\left[f_{1},f_{2}\right]$ (in Hz) using the band-wise root-mean-square (RMS) metric

(5) $${\mathrm{RMS}}_{B}\;=\;{\left(\frac{1}{N_{B}}\sum_{f\in B_{f}}|\bar{H}\left(f\right){|}^{2}\right)}^{1/2},$$

with $N_{B}$ the number of discrete frequency samples in the band. This scalar captures the average vibration level within the target range and is robust to narrow spectral features. Accordingly, the objective function minimized in this work is

(6) $$J\left(d\right)\;=\;{\mathrm{RMS}}_{B}\left(d\right),$$

where d collects the perforation diameters. Minimizing (5) drives down the overall FRF amplitude across the band of interest rather than at isolated frequencies, enabling frequency-selective attenuation suitable for finite plates with realistic boundary effects [24].

### 2.2. Particle Swarm Optimization (PSO)

Particle Swarm Optimization (PSO) is a population-based stochastic algorithm inspired by the collective movement of birds or fish schools. A swarm of $N_{p}$ particles explores the design space; each particle *i* is characterized by a position vector $x_{i}^{k}$ and a velocity vector $v_{i}^{k}$ at iteration *k*. The iterative update rules are

(7) $$\begin{array}{cc} v_{i}^{k+1} & =w_{I}\;v_{i}^{k}+c_{1}\;r_{1}\;\left(p_{i}-x_{i}^{k}\right)+c_{2}\;r_{2}\;\left(g-x_{i}^{k}\right),\;\mathrm{and} \end{array}$$

(8) $$\begin{array}{cc} x_{i}^{k+1} & =x_{i}^{k}+v_{i}^{k+1}, \end{array}$$

where $w_{I}$ is the inertia weight, $c_{1}$ and $c_{2}$ are the cognitive and social acceleration coefficients, $r_{1}$ and $r_{2}$ are random numbers uniformly distributed in [0,1], $p_{i}$ is the best position found so far by particle *i* (personal best), and g is the global best position found by the entire swarm.

The inertia term $w_{I}\;v_{i}^{k}$ governs the memory of previous motion, promoting exploration, while the cognitive and social terms direct each particle toward its own best experience and that of the group. This balance between exploration and exploitation allows PSO to converge efficiently toward a global optimum without requiring gradient information. Owing to its simplicity, small number of control parameters, and suitability for parallel computation, PSO has been widely applied to continuous optimization problems such as structural parameter tuning and metamaterial design [25,26].

**Figure 1:**
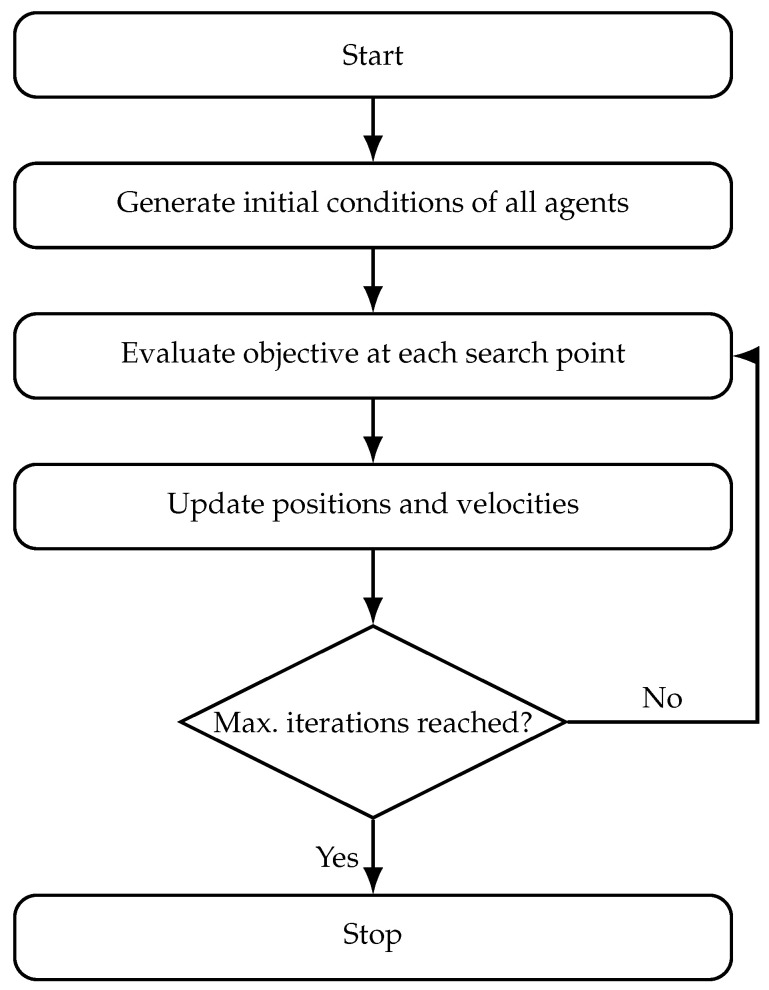
Flowchart of the Particle Swarm Optimization algorithm.

The main stages of the algorithm are summarized in the flowchart of Figure 1. Starting from a random initialization of the particle positions, the algorithm repeatedly evaluates the objective function, updates the personal and global best solutions, and modifies the particle velocities and positions until a stopping criterion (typically the maximum number of iterations) is satisfied.

**Figure 2:**
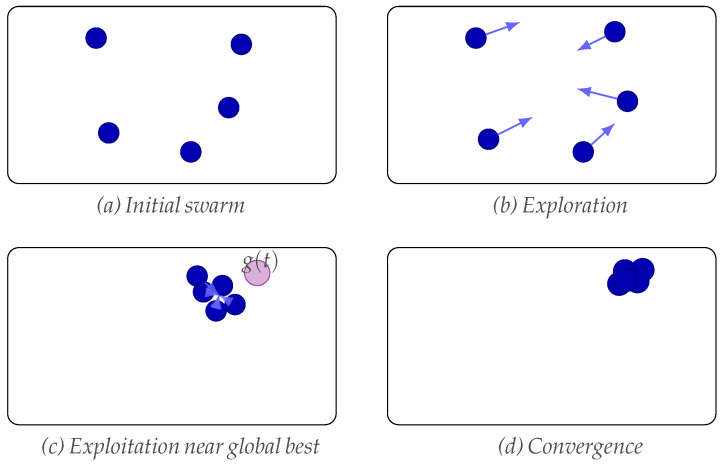
PSO progression in two rows: (**a**) initial swarm, (**b**) exploration, (**c**) exploitation near the global best, and (**d**) convergence at the same optimum.

To illustrate how the swarm actually evolves in the design space, Figure 2 shows a sequence of snapshots. Panel (a) depicts the random distribution of particles at the beginning of the process. During the early iterations the swarm explores the search space, as indicated by the arrows in panel (b). Once a promising region is detected, the particles exploit the neighborhood of the global best solution, clustering progressively around it as shown in panel (c), where the current global best g(t) is highlighted. Finally, the swarm converges and all particles collapse at the same location of the optimum (panel (d)), demonstrating the combined effect of cognitive and social acceleration in driving the population toward the global minimum.

## 3. Case Study and Problem Statement

**Figure 3:**
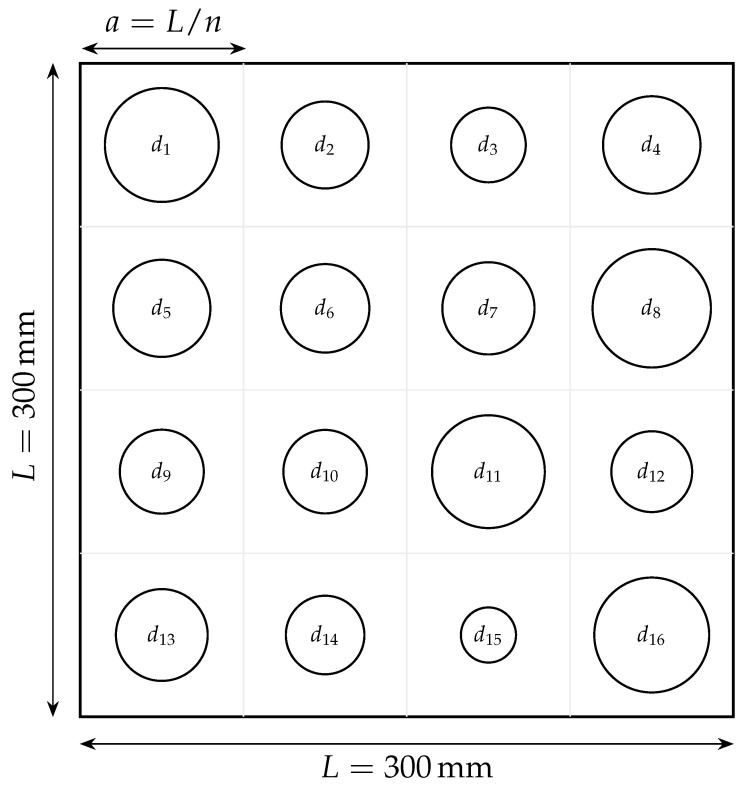
Illustrative finite panel (300×300 mm, h=3 mm) with a 4×4 grid of perforations. Each hole is a decision variable labeled $d_{1}$ to $d_{16}$. Diameters shown here are random purely for illustration.

We consider a finite square aluminum plate of side *L* = 300 mm and thickness h=3 mm, perforated with either a 4×4 or a 5×5 grid of circular holes. The center-to-center grid pitch is

$$a=\frac{L}{n}\;=\;\left\{\begin{array}{cc} 75\;\mathrm{mm}, & n=4,\\ 60\;\mathrm{mm}, & n=5, \end{array}\right.$$

with the grid centered on the plate. As represented in Figure 3, each circular hole is characterized by its diameter $d_{j}$, and the optimization treats the $n^{2}$ diameters as independent design variables collected in

$$d={\left[d_{1},\;d_{2},\;\ldots ,\;d_{n^{2}}\right]}^{\;⊤},\;n\in \left\{4,5\right\}.$$

To ensure manufacturability and avoid overlap, box constraints and a minimum ligament are enforced:

(9) $$d_{j}^{\min}\leq d_{j}\leq d_{j}^{\max},\;j=1,\ldots ,n^{2},$$

together with a minimum ligament distance $\delta_{\min}$ between adjacent holes and between edge holes and the plate boundary. With hole centers on a square grid of pitch *a*, choosing

(10) $$d_{j}^{\max}\;\leq \;a-2\;\delta_{\min}$$

is sufficient to satisfy both the no-overlap condition between neighbors and the edge-clearance requirement for border cells. Here we used $\delta_{\min}=5$ mm. The lower bound $d_{j}^{\min}$ can approach a small positive value (e.g., 0.2 mm) to represent an effectively solid cell when desired.

The design goal is to minimize the global vibration level within an application-driven frequency band $B_{f}=\left[f_{1},f_{2}\right]$. Let $H_{ik}\left(\omega \right)$ denote the single-input/single-output FRF magnitude (accelerance) from a force DOF *k* to response DOF *i*. With $N_{r}$ response locations, we define the location-averaged FRF magnitude as

(11) $$\bar{H}\left(f\right)\;=\;\frac{1}{N_{r}}\sum_{i=1}^{N_{r}}H_{ik^{*}}\left(2\pi f\right),$$

and the band-wise root-mean-square (RMS) metric

(12) $${\mathrm{RMS}}_{B}\;=\;{\left(\frac{1}{N_{B}}\sum_{f\in B_{f}}|\bar{H}\left(f\right){|}^{2}\right)}^{1/2},$$

where $N_{B}$ is the number of sampled frequencies in the band. The optimization problem is

(13) $$\begin{array}{cc} \min_{d}\;J\left(d\right)\; & ={\mathrm{RMS}}_{B_{f}}\left(d\right)\\ \mathrm{subject}\;\mathrm{to}:\;\; & d_{j}^{\min}\leq d_{j}\leq d_{j}^{\max},\;j=1,\ldots ,n^{2}, \end{array}$$

with $d_{j}^{\max}$ chosen such that $d_{j}^{\max}\leq a-2,\delta_{\min}$ [see Equation (10)]. This objective reduces the average FRF magnitude over the entire target band, yielding frequency-selective attenuation that is robust to narrow spectral features in finite plates.

The optimization focuses exclusively on vibration mitigation, without considering static or structural performance criteria. In particular, effects related to stress concentration, yielding, fatigue, or overall stability were not included in the optimization objective or constraints. All plates were modeled as linearly elastic thin structures undergoing small-amplitude vibrations, with the goal of minimizing dynamic response rather than ensuring load-carrying capacity. The free–free boundary conditions were adopted both numerically and experimentally, to replicate the suspension setup used in the roving-hammer tests. The excitation consisted of transient point forces normal to the plate surface, representing the hammer impacts applied during the experiments. Consequently, the optimization adjusted the hole diameters solely to minimize the vibration level within the target frequency bands, under the same material, geometry, and boundary conditions.

Problem (13) is solved with a Particle Swarm Optimization (PSO) scheme, using a swarm of $N_{p}=70$ particles and a maximum of $N_{\mathrm{it}}=100$ iterations with early stopping on convergence. Particle positions are initialized uniformly within $\left[d_{j}^{\min},d_{j}^{\max}\right]$ for all *j*. The inertia weight and acceleration coefficients followed conventional settings ($w_{I}=0.7$, $c_{1}=c_{2}=1.4$), which ensured stable convergence and consistent results across independent runs. The cost function J(d) was evaluated for each particle based on the finite-element frequency response within the prescribed band $B_{f}$. It should be noted that Figure 1 illustrates the generic workflow of the PSO algorithm, independent of the particular optimization problem. In the present study, the step labeled “Evaluate objective at each search point” corresponds specifically to computing the root-mean-square magnitude of the averaged frequency response function (FRF) within the target frequency band, as defined in Equations (11) and (12).

**Figure 4:**
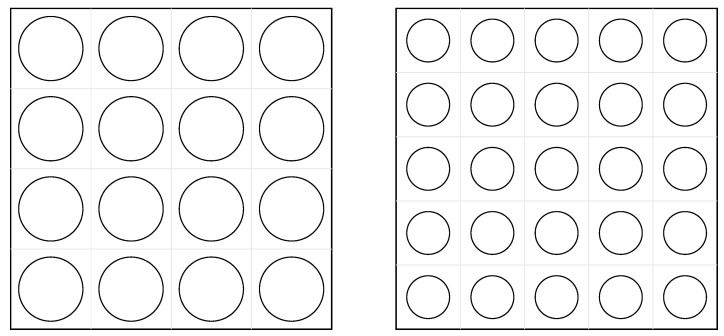
Uniform reference plates for validation: (**left**) 4×4 layout with d=60 mm in all cells; (**right**) 5×5 layout with d=40 mm in all cells.

To isolate the benefit of individually tuned diameters, two uniform reference plates are used: a 4×4 panel with d=60 mm in all cells and a 5×5 panel with d=40 mm in all cells (Figure 4).

## 4. Numerical Model

We modeled the square plate of side length L=300 mm and thickness h=3 mm in SDTools 6.2 2019 for MATLAB R2018b, using the built-in p_shell formulation. This element implements a Reissner–Mindlin (first-order shear deformation) plate/shell model, which includes transverse-shear effects and is suitable for thin to moderately thick plates.

The aluminum plate was modeled as a homogeneous, isotropic, and linearly elastic material with constant mechanical properties over the analyzed frequency range. The constitutive behavior follows the standard small-strain linear elastic model, characterized by an experimentally measured Young’s modulus E=72 GPa and a Poisson’s ratio ν=0.35. A uniform modal damping ratio of ξ=0.0018 was applied to all modes, with a mass density of ρ=2700 kg/m^3^. Further details on the experimental characterization of these parameters are provided in Section 5 and Section 6.

The perforations were represented explicitly in the finite-element mesh, and each unit cell contains a single circular cut-out whose diameter is treated as an independent design variable for the optimization. The finite-element assembly, boundary conditions, selection of excitation and response degrees of freedom, and the computation of frequency response functions (FRFs) were all implemented in MATLAB using the SDTools routines.

**Figure 5:**
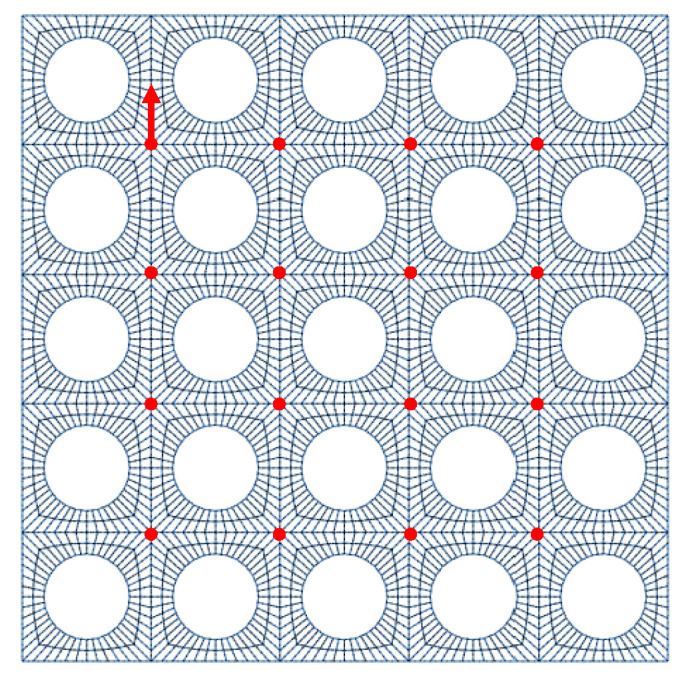
Finite-element mesh of the 5×5 perforated plate showing the fixed reference accelerometer location (red arrow) and the multiple impact points for the roving-hammer excitation (red dots).

The finite-element model reproduced the configuration shown in Figure 5, incorporating the fixed reference sensor location and the corresponding excitation points. This setup was used to compute a consistent set of frequency response functions (FRFs) with common reference and excitation positions, ensuring that the results remain independent of the perforation diameters and comparable across the different geometrical configurations considered during the optimization process.

For a given excitation degree of freedom *k* (impact point) and response degree of freedom *i* (accelerometer location), the single-input/single-output FRF $H_{ik}\left(\omega \right)$ is evaluated over the target frequency bands. To obtain a robust scalar measure per plate for the optimization objective, the magnitude of the FRFs from all response locations is averaged before computing the root-mean-square (RMS) value in each target frequency band. The FEM outputs were coupled with the PSO optimization loop to evaluate the RMS FRF amplitude over the prescribed frequency bands.

**Figure 6:**
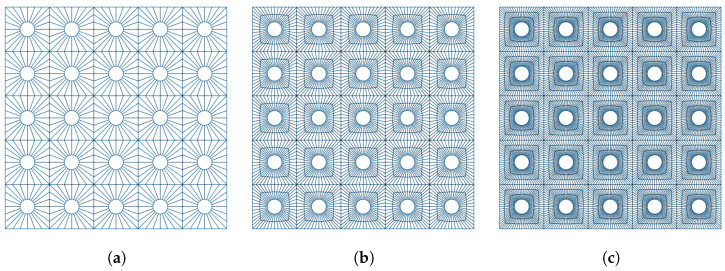
Finite-element meshes used in the refinement study. (**a**) Coarse mesh. (**b**) Medium mesh. (**c**) Fine mesh.

To verify the numerical convergence of the model, a mesh refinement analysis was conducted for the 5×5 perforated reference plate. Three meshes were generated with increasing local refinement. Representative wireframe views of the three meshes are shown in Figure 6.

**Figure 7:**
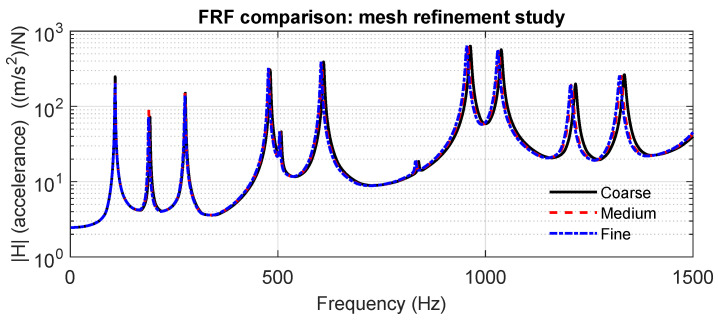
Comparison of averaged FRFs for the coarse, medium, and fine meshes. Minimal differences between the medium and fine meshes indicate numerical convergence.

The averaged frequency response functions (FRFs) obtained from the coarse, medium, and fine meshes are compared in Figure 7. All three meshes lead to practically identical results in the low-frequency range, including the first resonance modes, indicating that the global dynamic behavior is already well captured with the coarser discretization. Small differences appear only at higher frequencies (above approximately 800–900 Hz), where the finer geometric resolution slightly improves the accuracy of the local deformation fields around the perforation edges. However, the medium and fine meshes yield nearly indistinguishable FRFs over the full frequency range, with differences below 2%. For this reason, the medium mesh was adopted in all subsequent simulations, as it provides mesh-independent results while maintaining a reasonable computational cost.

**Figure 8:**
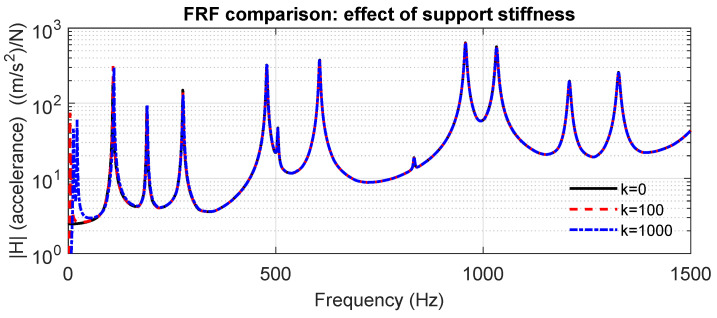
Effect of support stiffness on the averaged FRF. Differences are concentrated in the rigid-body region below 50 Hz.

Although the numerical model assumes nominally free–free boundary conditions, in the experiment the plate is suspended using elastic supports. To assess the influence of this practical boundary condition, elastic springs were added at four corner locations in the numerical model, connecting the out-of-plane displacement DOF to ground. Four stiffness levels were examined (k=0, 100, and 1000 N/m), where k=0 represents a fully free condition (no spring) and the other values span the range of soft textile or rubber bands typically used in suspended setups. The resulting averaged FRFs (Figure 8) show that the spring stiffness mainly affects the low-frequency range corresponding to rigid-body motions, shifting the first resonance by up to 1–8 Hz. However, for frequencies above 50 Hz, the effect of the boundary elasticity becomes negligible, and the FRF curves collapse for all tested stiffness values. Therefore, the free–free model provides an adequate representation for the frequency ranges analyzed in this study, and no boundary compliance correction is needed in the optimization phase.

## 5. Experimental Methodology

The experimental campaign aimed to validate the numerical predictions of vibration attenuation in perforated aluminum plates. The work comprised three main stages: characterization of the base material, fabrication of the panels, and dynamic testing through roving-hammer impact measurements.

### 5.1. Material Characterization

**Figure 9:**
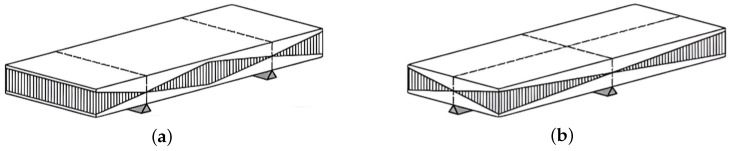
Experimental setups used for the ASTM E1876 tests: (**a**) flexural (bending) resonance and (**b**) torsional resonance.

To parameterize the finite-element model, the elastic modulus *E*, Poisson’s ratio ν, and modal damping ratio ζ of the aluminum material were determined experimentally. A rectangular aluminum coupon (150 mm × 55 mm × 3 mm) was tested following the ASTM E1876 standard [27]. Flexural and torsional resonance frequencies were extracted from impact-hammer frequency response functions (FRFs), and the vibrations were measured using a microphone positioned near the specimen, as illustrated in Figure 9.

Following ASTM E1876, the elastic constants were computed from the measured resonance frequencies using

(14) $$E=0.9465\left(\frac{m\;f^{2}}{w}\right)\left(\frac{L^{3}}{t^{3}}\right)T,$$

(15) $$G=\frac{4\;L\;m\;f_{t}^{2}}{w\;t}\;R,$$

(16) $$\nu =\frac{E}{2\;G}-1,$$

where *m* is the specimen mass, *f* and $f_{t}$ are the flexural and torsional natural frequencies, *w* is the specimen width, *L* is its length, *t* is its thickness, and *T* and *R* are the geometry correction factors specified in the ASTM E1876 standard. The modal damping ratio ζ was later obtained from the half-power bandwidth method applied to the first bending resonance of the flexural FRF.

### 5.2. Fabrication of the Panels

Perforated plates were manufactured from 3 mm thick 1100-H14 aluminum sheets (commercial stock material, no specific manufacturer) using a CNC milling machine Tormach 1100M (Tormach Inc., Waunakee, WI, USA). All plates share the same outer dimensions (300 × 300 mm^2^) and thickness (3 mm). Three optimized layouts—16 holes (target band 200–400 Hz) and 25 holes optimized for 600–800 Hz and 500–1000 Hz—were produced together with uniform-hole reference plates for each configuration. For consistency, the reference plates used uniform perforations of 60 mm diameter in the 4 × 4 case and uniform perforations of 40 mm diameter in the 5 × 5 case. The two reference plates used in validation are shown in Figure 4.

**Figure 10:**
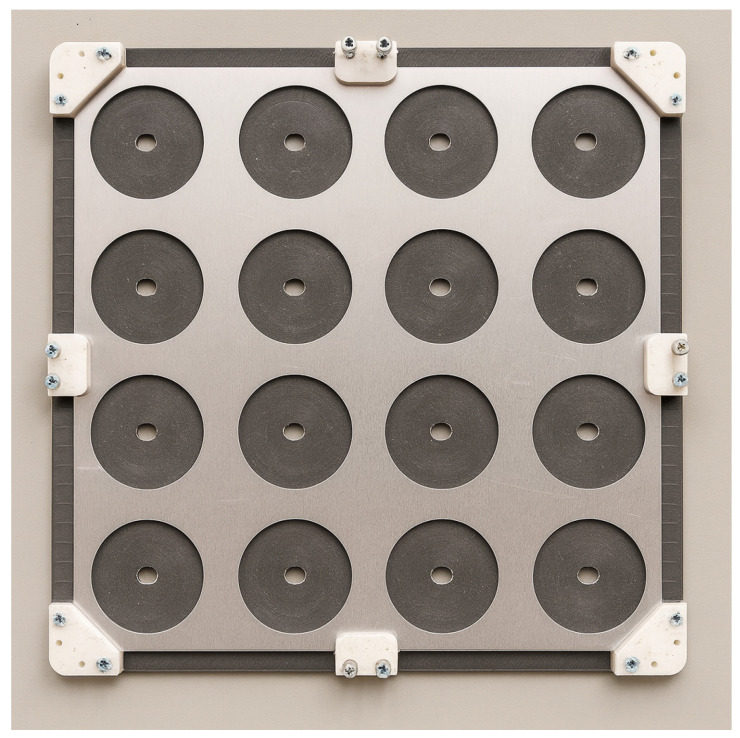
Aluminum perforated panel mounted on the CNC machine after the drilling process. The image shows the 4×4 array of perforations and the clamping system used to hold the plate during machining.

Dimensional tolerances of the perforation diameters were within ±0.2 mm, ensuring consistency with the numerical models. An example of a finished plate still mounted on the CNC table, showing the 4 × 4 array of perforations and the clamping system used during machining, is presented in Figure 10.

### 5.3. Dynamic Testing of Perforated Panels

**Figure 11:**
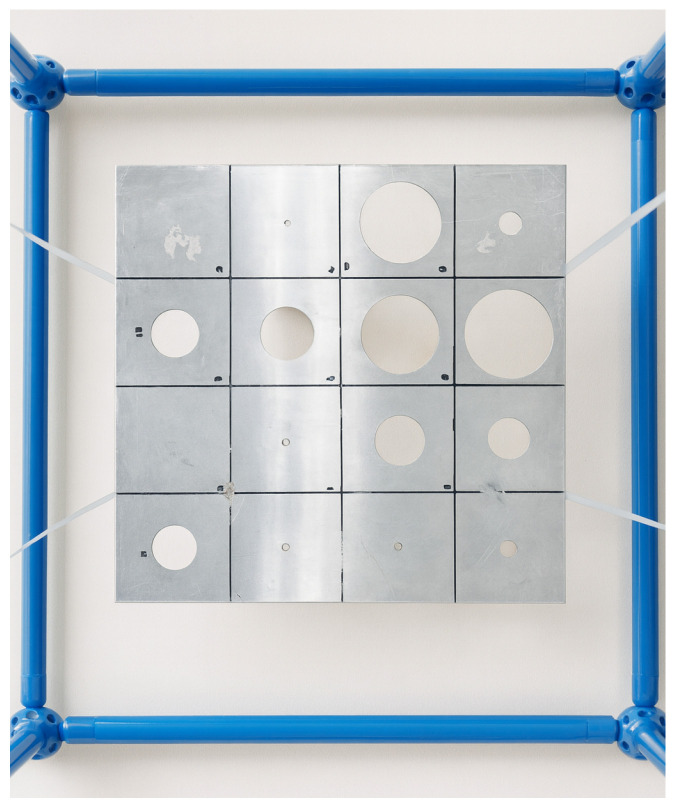
Experimental setup for the dynamic tests. The perforated aluminum panel is suspended with elastic cords to approximate free–free boundary conditions during the roving-hammer measurements.

The vibration response of the fabricated plates was measured using a roving-hammer technique to obtain frequency response functions (FRFs). Each plate was suspended with elastic cords to approximate free–free boundary conditions, as illustrated in Figure 11. A single accelerometer was kept fixed at the reference position marked in red in Figure 5, while the instrumented modal hammer excited successively the red impact points. Each FRF was measured five times and the results were averaged to reduce the effects of experimental noise and improve repeatability. The excitation and acquisition were performed in the frequency range from 0 to 2560 Hz, with a frequency resolution of 0.625 Hz, ensuring sufficient spectral detail to identify the principal resonances of the perforated panels. This configuration ensured a consistent reference sensor and accurate estimation of mobility FRFs across the panel surface. The averaged FRFs were then used to obtain the global dynamic response of each panel.

The finite-element model reproduced both the fixed accelerometer location and the distribution of hammer impact points to allow direct comparison between simulated and experimental FRFs.

## 6. Results

This section presents the results of the study, including the outcomes of the material characterization tests, the numerical optimization of the perforated panels, and the experimental validation through impact testing. Comparisons between numerical predictions and measured frequency response functions (FRFs) are provided to assess the effectiveness of the proposed optimization strategy.

### 6.1. Material Characterization Results

The mechanical properties of the aluminum alloy 1100-H14 used for all panels were obtained experimentally following the ASTM E1876 standard, as described in Section 5. The flexural and torsional natural frequencies were extracted from impact-hammer frequency response functions (FRFs) and used in Equations (14)–(16) to estimate the elastic modulus, shear modulus, and Poisson ratio. The results, summarized in Table 1, were consistent with literature values for this alloy. The modal damping ratio ζ was determined from the half-power bandwidth method applied to the first bending mode.

**Figure 12:**
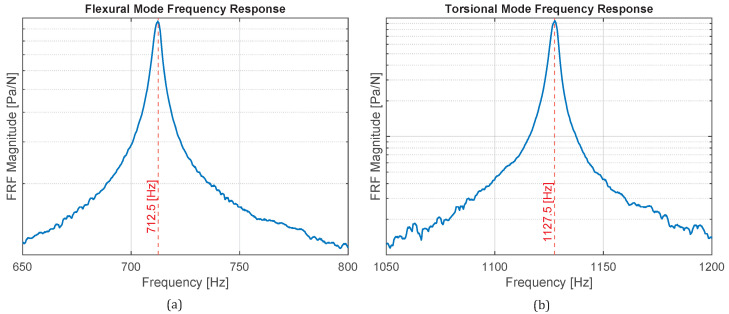
Measured frequency response functions (FRFs) obtained from the impact-hammer tests for the aluminum specimen: (**a**) flexural and (**b**) torsional vibration modes. The blue curves correspond to the measured accelerance FRFs, while the red dashed lines indicate the experimentally identified resonance frequencies for each mode.

Figure 12 shows the measured frequency response functions (FRFs) obtained from the impact hammer tests. Figure 12a corresponds to the flexural mode, while Figure 12b shows the torsional response. The fundamental resonances used to estimate the elastic and shear moduli are clearly visible in both curves.

**Figure 13:**
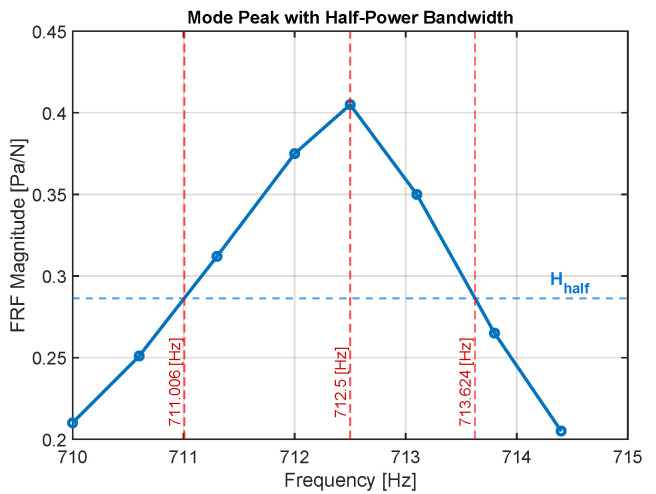
Estimation of the modal damping ratio ζ using the half-power bandwidth method applied to the first flexural FRF peak. The frequencies $f_{1}$ and $f_{2}$ correspond to the half-power points ($H_{\mathrm{half}}$) on each side of the resonance.

To estimate the modal damping ratio of the aluminum, the half-power bandwidth method was applied to the flexural FRF. The resonant peak corresponding to the first bending mode was bounded by the half-power limits ($H_{\mathrm{half}}$), defined as the frequencies at which the amplitude drops to $1/\sqrt{2}$ of the peak value. Since the data were recorded as discrete samples, the limits were obtained by linear interpolation between the closest data points on both sides of the resonance. The resulting frequencies $f_{1}=710.98\;\mathrm{Hz}$ and $f_{2}=713.60\;\mathrm{Hz}$ yield an experimental damping ratio of ζ=0.0018. Figure 13 illustrates this procedure.

### 6.2. Numerical Optimization Results

**Figure 14:**
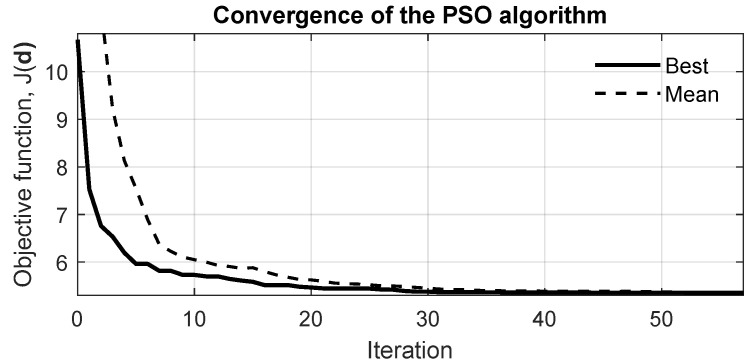
Convergence history of the Particle Swarm Optimization (PSO) algorithm. The best and mean objective values (J(d)) correspond to the band-averaged RMS magnitude of the averaged FRF within the target frequency range.

The optimization was implemented in MATLAB using the built-in particleswarm solver and coupled to the FEM routines (SDTools) that evaluate the objective function J(d) as the band-averaged RMS of the location-averaged FRF. At each PSO iteration, candidate diameter vectors are generated, projected onto the feasible box $\;[d_{j}^{\min},d_{j}^{\max}]$ (with the ligament constraint enforced by the choice of $d_{j}^{\max}$), and passed to the FE model to compute the FRFs on a uniform frequency grid (1 Hz resolution over the analyzed range). The magnitudes are averaged across response locations and the RMS is taken over the target band $B_{f}$ to produce J(d). The PSO and simulation parameters used in the study are summarized in Table 2. Convergence was monitored via the built-in tolerance on improvement and by the evolution of the global best objective (Figure 14).

Before analyzing the three validated study cases, a preliminary optimization campaign was conducted to evaluate the general behavior of the proposed approach across a broad set of target frequency ranges. Four independent optimization problems were solved for the 5×5 perforated plate, targeting the bands 100–400 Hz, 400–800 Hz, 800–1200 Hz, and 1200–1600 Hz. This analysis provides an expanded view of how the PSO-driven diameter tuning reshapes the dynamic response of the plate to selectively attenuate vibration.

Figure 14 shows the convergence history of the PSO algorithm for a representative case. The best and mean objective values, corresponding to the band-averaged RMS magnitude of the frequency response function (FRF), are plotted as a function of iteration. A rapid reduction in both metrics is observed during the first 20 iterations, followed by a gradual stabilization of the global best value, indicating convergence of the swarm and consistent optimization performance.

**Figure 15:**
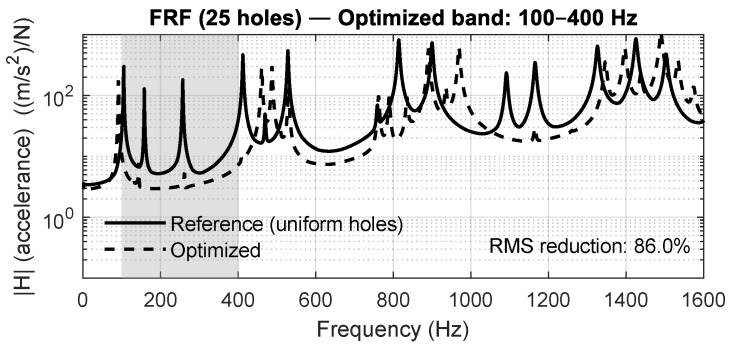
Averaged FRF for the 5×5 panel optimized in the 100–400 Hz band. A clear attenuation of vibration amplitude is observed inside the target frequency range.

**Figure 16:**
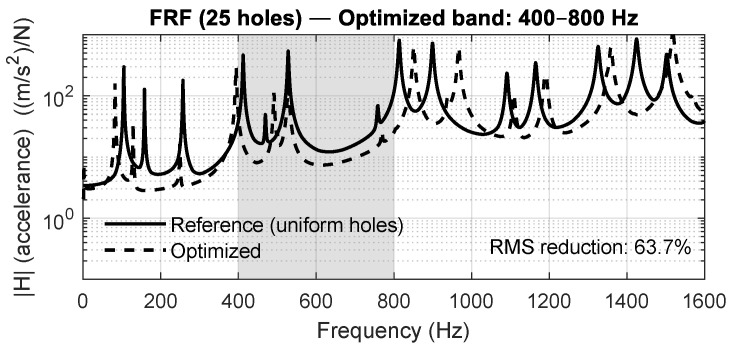
Averaged FRF for the 5×5 panel optimized in the 400–800 Hz band.

**Figure 17:**
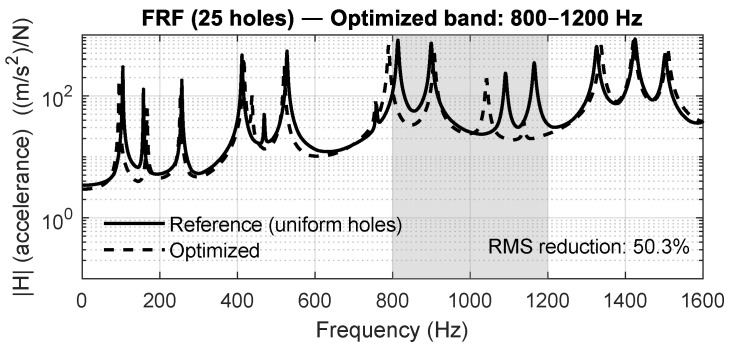
Averaged FRF for the 5×5 panel optimized in the 800–1200 Hz band.

**Figure 18:**
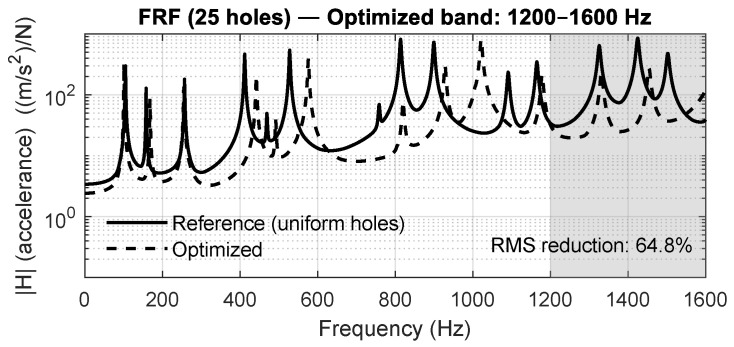
Averaged FRF for the 5×5 panel optimized in the 1200–1600 Hz band.

Figure 15, Figure 16, Figure 17 and Figure 18 show the averaged FRFs obtained for each optimization band. In all cases, a clear attenuation of vibration amplitude is observed within the targeted frequency intervals, demonstrating the repeatability and bandwidth flexibility of the optimization method. Depending on the band, the RMS reduction inside the target interval ranges between 50% and 86%, confirming that the optimization process drives energy away from the specified frequency region.

**Figure 19:**
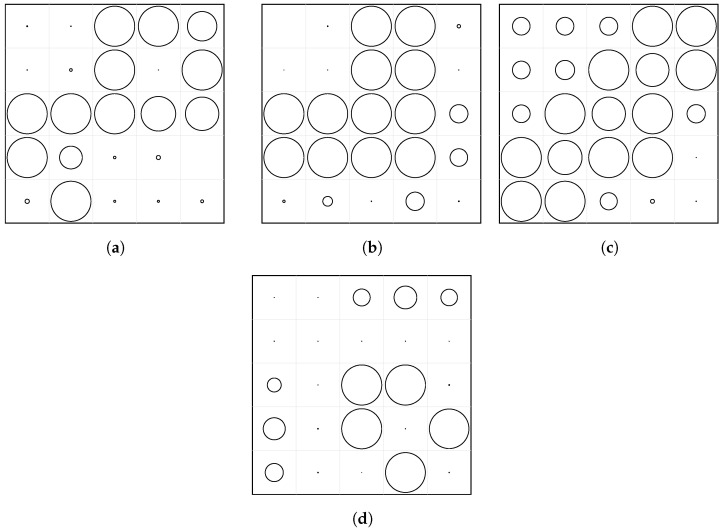
Optimized perforation layouts for the 5×5 plate obtained using PSO for four different target frequency bands. Circle size is proportional to the final hole diameter. (**a**) 100–400 Hz. (**b**) 400–800 Hz. (**c**) 800–1200 Hz. (**d**) 1200–1600 Hz.

The optimized perforation layouts for these four frequency bands are presented in Figure 19. Low-frequency targets tend to produce more asymmetric layouts, with clusters of large perforations forming in regions associated with higher modal curvature. Conversely, higher-frequency targets often yield more structured patterns, frequently exhibiting partial symmetry along one or two plate axes, consistent with the increased modal complexity at higher frequencies. These observations confirm that the optimization algorithm adapts the stiffness distribution in a physically interpretable way, creating stiffer or more compliant regions depending on the modal shapes governing the target band.

The optimization process was then carried out for the three main configurations analyzed and experimentally validated in this study: (1) a 4×4 grid optimized in the 200–400 Hz band, (2) a 5×5 grid optimized in the 600–800 Hz band, and (3) a 5×5 grid optimized in the 500–1000 Hz band. For each configuration, the PSO algorithm adjusted the individual hole diameters to minimize the root-mean-square (RMS) magnitude of the averaged FRF within the target frequency range.

The optimization process was performed for three perforated configurations: (1) a 4×4 grid optimized in the 200–400 Hz band, (2) a 5×5 grid optimized in the 600–800 Hz band, and (3) a 5×5 grid optimized in the 500–1000 Hz band. For each configuration, the Particle Swarm Optimization (PSO) algorithm adjusted the individual hole diameters to minimize the root-mean-square (RMS) magnitude of the averaged FRF within the target frequency range.

**Figure 20:**
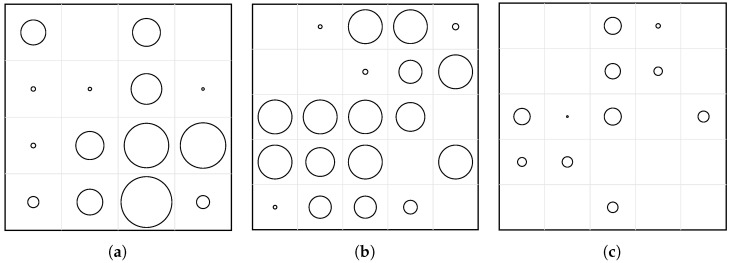
Optimized perforation layouts obtained from PSO for the three configurations. Each circle corresponds to a perforation with diameter d>3 mm. (**a**) Case A: 4×4 panel optimized in 200–400 Hz. (**b**) Case B: 5×5 panel optimized in 600–800 Hz. (**c**) Case C: 5×5 panel optimized in 500–1000 Hz.

The optimized perforation layouts obtained for the three configurations are shown in Figure 20. Circles represent the perforations, drawn only when the final diameter is larger than 3 mm; smaller values were neglected and treated as solid regions. The resulting geometries exhibit clear non-uniformity: clusters of large perforations appear in regions of high modal displacement, while smaller or absent perforations form stiffer zones that shift the modal energy away from the target frequency bands.

**Figure 21:**
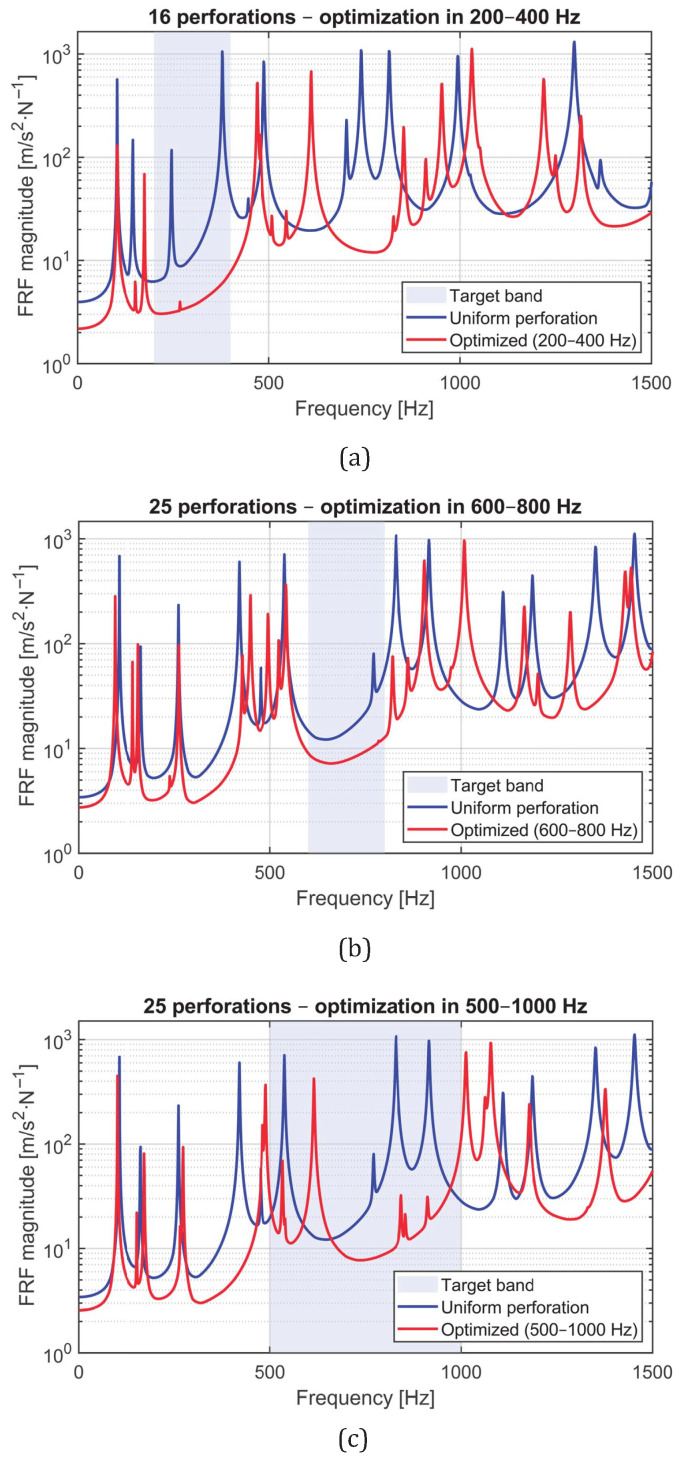
Comparison between experimental FRFs of uniform and optimized panels. Each plot corresponds to one optimization case: (**a**) 4×4 panel optimized in 200–400 Hz, (**b**) 5×5 panel optimized in 600–800 Hz, and (**c**) 5×5 panel optimized in 500–1000 Hz. The shaded regions correspond to the optimization bands, where a significant attenuation of vibration amplitude is observed.

The numerical and experimental FRFs of the optimized and uniform plates for the three configurations are compared in Figure 21. In all cases, the optimized designs exhibit a pronounced reduction in vibration amplitude within the targeted frequency bands, confirming that the PSO-based diameter tuning effectively induces local dynamic effects that suppress structural response. Outside these bands, both configurations show similar behavior, indicating that the optimization modifies the dynamic response in a localized and controlled manner.

Quantitatively, the RMS of the averaged FRF within the target band was reduced as summarized in Table 3. The largest improvement corresponds to the 4×4 configuration (Case A), which achieved a reduction of approximately 96.5% in the RMS amplitude compared to the uniform plate. The 5×5 configurations optimized in higher frequency bands showed reductions of 63.6% and 73.2%, respectively. These results confirm that the PSO framework effectively redistributes perforations to minimize dynamic response while maintaining the same mass and boundary conditions.

### 6.3. Experimental Validation and Comparison

**Figure 22:**
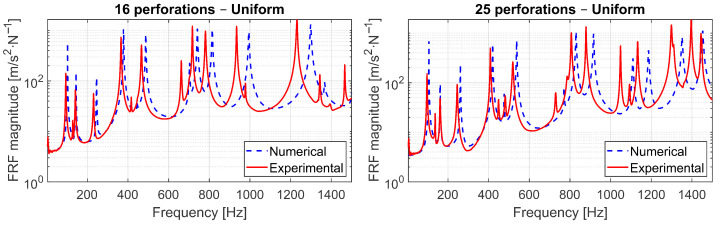
Comparison between numerical and experimental FRFs for the uniform perforated panels: (**left**) 16-hole configuration and (**right**) 25-hole configuration.

The experimental tests were conducted on the fabricated aluminum panels to validate the numerical predictions. Figure 22 compares the measured and simulated frequency response functions (FRFs) for the uniform perforation configurations with 16 and 25 holes. A strong agreement was obtained between numerical and experimental results, confirming that the finite-element model accurately reproduces the dynamic behavior of the perforated plates across the investigated frequency range.

A small frequency shift can be observed, with the numerical peaks slightly displaced toward higher frequencies, and the deviation increasing with mode order. This trend is attributed to a marginally stiffer numerical model. Such differences are expected in experimental validations of thin plates and reflect typical modeling simplifications.

**Figure 23:**
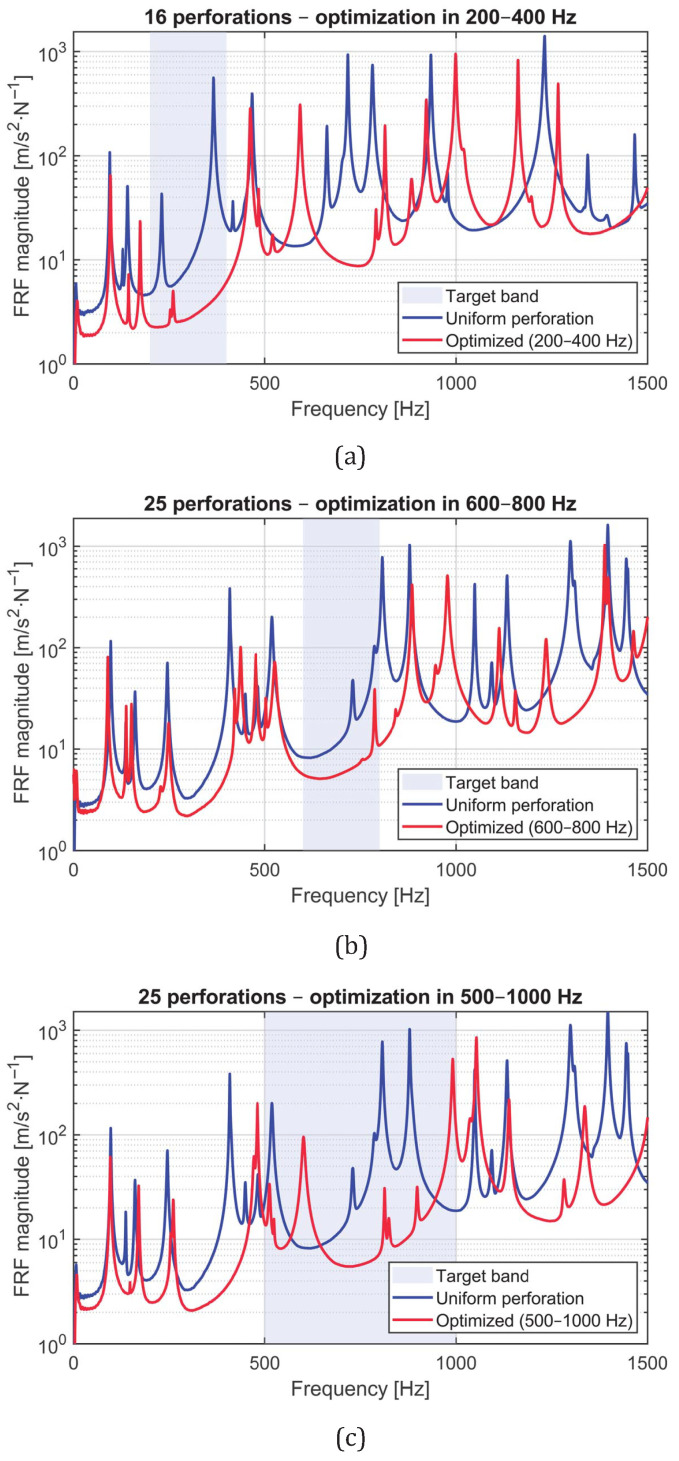
Experimental FRFs of uniform (blue) and optimized (red) panels for the three cases: (**a**) 16 perforations optimized in 200–400 Hz, (**b**) 25 perforations optimized in 600–800 Hz, and (**c**) 25 perforations optimized in 500–1000 Hz. The shaded regions indicate the target optimization bands.

Subsequently, the optimized and uniform panels were experimentally compared to assess the effectiveness of the optimization process. Figure 23a–c show the experimental FRFs for each configuration: (a) 16 holes optimized in the 200–400 Hz band, (b) 25 holes optimized in the 600–800 Hz band, and (c) 25 holes optimized in the 500–1000 Hz band. In all cases, the optimized plates exhibit a clear attenuation of vibration amplitude within the target frequency bands, consistent with the numerical predictions.

Quantitatively, the reduction in the RMS magnitude of the FRF within the optimized frequency bands was computed for each configuration. The results, summarized in Table 4, confirm the high efficiency of the Particle Swarm Optimization (PSO) approach when applied to experimental conditions, with reductions comparable to the numerical predictions.

## 7. Discussion

The results demonstrate that optimizing each perforation individually provides an effective and flexible means to achieve frequency-selective vibration attenuation in lightweight plates. Compared with traditional designs based on uniform or periodic perforations, the proposed approach allows fine tuning of the dynamic response, leading to a significant reduction in vibration amplitudes within prescribed bands while maintaining the same overall mass and geometry. The experimental validation confirmed these findings, with RMS reductions between 51% and 95% depending on the configuration and frequency range. From a physical perspective, the individually optimized layouts generate a spatially heterogeneous stiffness field that modifies the coupling between neighboring regions of the plate. This nonuniform stiffness distribution produces local impedance contrasts and partial wave reflections, mechanisms that can suppress bending wave propagation and concentrate modal energy away from the target frequency bands. Unlike periodic phononic structures, where bandgaps emerge due to translational symmetry and Bragg scattering, the present approach achieves similar attenuation through an aperiodic yet physically consistent configuration of perforations. This result suggests that precise control of local compliance, rather than strict periodicity, is sufficient to induce localized vibration suppression in thin plates.

The optimized perforation layouts (Figure 20) exhibit characteristic spatial patterns that depend on the target frequency band. In the low-frequency case (200–400 Hz, Case A), the algorithm favored larger perforations clustered toward one corner of the plate, with smaller holes or solid regions along the opposite diagonal. This asymmetry increases local flexibility in regions of high modal curvature, effectively shifting the first bending modes to higher frequencies. In the mid- and high-frequency cases (600–800 Hz and 500–1000 Hz, Cases B and C), the optimized patterns become more evenly distributed and show partial symmetry with respect to the plate diagonal. This quasi-symmetric arrangement results in a more balanced stiffness field, reducing modal coupling and ensuring attenuation across several higher-order modes.

When comparing the numerical and experimental FRFs, an excellent correspondence is observed in the low–frequency range, confirming the predictive capability of the finite-element model and the adequacy of the identified damping ratio. At higher frequencies, small differences appear in the spacing and amplitude of resonant peaks (Figure 22). The mesh refinement analysis demonstrated that these discrepancies are not associated with discretization, since the medium and fine meshes yield virtually identical FRFs. Likewise, the boundary compliance study showed that the suspended support condition only affects the rigid-body region below 50 Hz, with negligible influence in the frequency bands of interest. Therefore, the remaining deviations can be primarily attributed to (i) uncertainties in the material properties of the manufactured plate and (ii) the idealizations inherent to the plate formulation (e.g., Reissner–Mindlin kinematics, constant thickness, and perfectly circular perforations). Minor geometric tolerances introduced during machining and small mass loading effects from the accelerometer may further contribute to the observed differences. Despite these factors, the agreement remains strong across the operational frequency bands relevant to the optimization.

The results also indicate that the observed vibration reduction is primarily associated with changes in stiffness distribution rather than simple mass effects. In fact, the optimized plates exhibit a slightly higher total mass than the uniform reference plates, since the optimization process tends to enlarge certain perforations while leaving others unperforated, leading to a net material increase. This finding confirms that the attenuation mechanism is fundamentally structural-dynamic—driven by controlled stiffness gradients and modal interference rather than a consequence of mass reduction.

The present work focused exclusively on vibration mitigation through geometric optimization of perforation diameters under fixed free–free boundary conditions. Nevertheless, the proposed methodology can be readily extended to include additional geometric and boundary parameters, such as plate thickness, hole arrangement, or support conditions, enabling a more comprehensive parametric exploration of their influence on dynamic behavior. Furthermore, future studies could couple the present vibration-based objective with structural criteria such as stress concentration, stiffness retention, or fatigue life, thereby establishing a multi-objective optimization framework that balances vibration suppression and mechanical strength. Such an extension would allow assessing how the perforation pattern affects both the dynamic response—quantified by the root-mean-square amplitude of the frequency response function—and the static performance of the plate.

## 8. Conclusions

This work presented a combined numerical–experimental framework for the design and validation of finite perforated cellular panels for vibration mitigation. Unlike most previous studies, which focus on infinitely periodic or idealized unit-cell models, the proposed approach considers finite plates and individually optimizes the diameter of each perforation, enabling precise control of the structural dynamic response within specific frequency bands.

Finite element models were integrated with a Particle Swarm Optimization (PSO) algorithm to minimize the root-mean-square amplitude of the frequency response function (FRF) in predefined frequency ranges. The optimized aluminum panels were subsequently fabricated and experimentally tested, showing strong agreement with numerical predictions. Reductions of up to 90% in vibrational amplitude were achieved within the targeted bands, confirming the effectiveness of the optimization strategy and validating the predictive capability of the numerical model.

The results demonstrate that individualized perforation optimization provides a lightweight, efficient, and scalable solution for vibration suppression, outperforming conventional uniform designs. By tailoring the stiffness and mass distribution through local geometric tuning, it is possible to achieve selective attenuation without adding external damping materials or increasing structural mass. While this study focused on aluminum plates with circular perforations and free–free boundary conditions, the methodology can be readily extended to other materials, non-circular geometries, and different boundary configurations.

Overall, the proposed framework establishes a practical and computationally efficient route for the design of advanced perforated panels with frequency-selective vibration control.

## Figures and Tables

**Table 1:** Material properties of the 1100-H14 aluminum plate obtained from ASTM E1876 tests.

Property	Symbol	Value
Young’s modulus	*E*	72GPa
Poisson ratio	ν	0.35
Modal damping ratio	ζ	0.0018

**Table 2:** PSO and simulation settings used in MATLAB.

Setting	Value/Description
Solver	particleswarm (MATLAB)
Swarm size	70 (SwarmSize = 70)
Max. iterations	100 (MaxIterations = 100)
Stopping tolerance	TolFun = $10^{-3}$ (relative improvement)
Inertia/accelerations	$w_{I}=0.7,\;c_{1}=c_{2}=1.4$
Initialization	Uniform in $\left[d_{j}^{\min},d_{j}^{\max}\right]$ for all *j*
Constraint handling	Box projection; ligament via $d_{j}^{\max}\leq a-2\delta_{\min}$
Objective J(d)	RMS of $\bar{H}\left(f\right)$ over $B_{f}$ (Equation (12))
FRF evaluation	1 Hz resolution over [0, 1600 Hz]; accelerance; average over sensors
Reporting	Best solution at termination; convergence curve in Figure 14

**Table 3:** Reduction in the RMS objective function J(d) compared with the uniform plate for each optimization case.

Configuration	Frequency Band [Hz]	Reduction (%)
4×4 perforations	200–400	96.5
5×5 perforations	600–800	63.6
5×5 perforations	500–1000	73.2

**Table 4:** Experimental reduction of the RMS magnitude within the target optimization bands, comparing uniform and optimized panels.

Configuration	Target Band [Hz]	RMS Reduction (%)
16 perforations	200–400	95.4
25 perforations	600–800	78.2
25 perforations	500–1000	51.5

## Data Availability

The original contributions presented in this study are included in the article. Further inquiries can be directed to the corresponding author.

## Nomenclature

**Symbol**	**Unit**	**Description**
*L*	mm	Plate side length
*h*	mm	Plate thickness
*a*	mm	Hole grid pitch (a=L/n)
*n*	–	Number of grid divisions per side (n∈{4,5})
$d_{j}$	mm	Diameter of perforation *j* (design variable)
d	mm	Vector collecting all perforation diameters
$\delta_{\min}$	mm	Minimum ligament between holes and plate edges
*m*	kg	Specimen mass (ASTM E1876 coupon)
*w*	mm	Specimen width (ASTM E1876 coupon)
*t*	mm	Specimen thickness (ASTM E1876 coupon)
*E*	GPa	Young’s modulus
*G*	GPa	Shear modulus
ν	–	Poisson ratio
ρ	kg/m^3^	Mass density
u(t)	m	Displacement vector in the time domain
a(t)	m/s^2^	Acceleration vector in the time domain
F(t)	N	External force vector in the time domain
$a_{i}\left(t\right)$	m/s^2^	Acceleration at response DOF *i*
$F_{k}\left(t\right)$	N	Excitation force applied at DOF *k*
${\hat{u}}_{i}\left(\omega \right)$	m	Fourier transform of the displacement at DOF *i*
${\hat{a}}_{i}\left(\omega \right)$	m/s^2^	Fourier transform of the acceleration at DOF *i*
${\hat{F}}_{k}\left(\omega \right)$	N	Fourier transform of the excitation force at DOF *k*
M	–	Mass matrix
C	–	Damping matrix
K	–	Stiffness matrix
Z(ω)	–	Dynamic stiffness matrix
$H_{ik}\left(\omega \right)$	(m/s^2^)/N	Accelerance FRF between excitation DOF *k* and response DOF *i*
$\bar{H}\left(f\right)$	(m/s^2^)/N	Averaged FRF magnitude across response locations
J(d)	(m/s^2^)/N	Objective function (band RMS of $\bar{H}$)
$N_{r}$	–	Number of response locations (sensors)
$N_{B}$	–	Number of frequency samples in a band
*f*	Hz	Frequency
$f_{1},\;f_{2}$	Hz	Lower and upper limits of the target frequency band
$f_{t}$	Hz	Torsional resonance frequency (ASTM E1876)
T,R	–	ASTM E1876 geometry correction factors
$N_{p}$	–	Number of particles in the PSO swarm
$N_{\mathrm{it}}$	–	Maximum number of PSO iterations
$x_{i}$	–	Position (design) vector of particle *i*
$v_{i}$	–	Velocity vector of particle *i*
$p_{i}$	–	Best position found by particle *i* (personal best)
g	–	Global best position found by the swarm
$c_{1},\;c_{2}$	–	Cognitive and social acceleration coefficients (PSO)
$r_{1},\;r_{2}$	–	Uniform random numbers in [0,1] (PSO)
ω	rad/s	Angular frequency (ω=2πf)
*j*	–	Imaginary unit ($j=\sqrt{-1}$)
ξ	–	Modal damping ratio (FE model)
ζ	–	Experimental damping ratio (half-power bandwidth)
$w_{I}$	–	Inertia weight (PSO)
$B_{f}=\left[f_{1},f_{2}\right]$	Hz	Target frequency band for optimization
${\mathrm{RMS}}_{B}$	–	Root-mean-square value within $B_{f}$
ASTM	–	American Society for Testing and Materials
CNC	–	Computer Numerical Control
DOF	–	Degree of Freedom
FEM	–	Finite Element Method
FRF	–	Frequency Response Function
PSO	–	Particle Swarm Optimization
RMS	–	Root Mean Square
SISO	–	Single-Input/Single-Output
